# Improving analytical reasoning and argument understanding: a quasi-experimental field study of argument visualization

**DOI:** 10.1038/s41539-018-0038-5

**Published:** 2018-12-04

**Authors:** Simon Cullen, Judith Fan, Eva van der Brugge, Adam Elga

**Affiliations:** 1 Department of Philosophy, Princeton University, Princeton, NJ USA; 2 Department of Philosophy, Carnegie Mellon University, Pittsburgh, PA USA; 3 Department of Psychology, Stanford University, Stanford, CA USA; 4 School of Historical and Philosophical Studies, University of Melbourne, Parkville, VIC Australia

## Abstract

The ability to analyze arguments is critical for higher-level reasoning, yet previous research suggests that standard university education provides only modest improvements in students’ analytical-reasoning abilities. What pedagogical approaches are most effective for cultivating these skills? We investigated the effectiveness of a 12-week undergraduate seminar in which students practiced a software-based technique for visualizing the logical structures implicit in argumentative texts. Seminar students met weekly to analyze excerpts from contemporary analytic philosophy papers, completed argument visualization problem sets, and received individualized feedback on a weekly basis. We found that seminar students improved substantially more on LSAT Logical Reasoning test forms than did control students (*d* = 0.71, 95% CI: [0.37, 1.04], *p* < 0.001), suggesting that learning how to visualize arguments in the seminar led to large generalized improvements in students’ analytical-reasoning skills. Moreover, blind scoring of final essays from seminar students and control students, drawn from a parallel lecture course, revealed large differences in favor of seminar students (*d* = 0.87, 95% CI: [0.26, 1.48], *p* = 0.005). Seminar students understood the arguments better, and their essays were more accurate and effectively structured. Taken together, these findings deepen our understanding of how visualizations support logical reasoning and provide a model for improving analytical-reasoning pedagogy.

## Short heading: Argument-visualization training leads to improved reasoning skills

The ability to analyze arguments is critical for higher-level reasoning, yet previous research suggests that standard university education provides only modest improvements in students’ analytical reasoning abilities. What pedagogical approaches are most effective for cultivating these skills? We investigated the effectiveness of a 12-week undergraduate seminar in which students learned to visualize the logical structures implicit in argumentative texts. We found that students who completed this seminar improved substantially more on LSAT Logical Reasoning test forms than did control students, showing that this intervention produced generalized improvements in analytical reasoning skills. Moreover, seminar students wrote more accurate and effectively structured final essays than did control students. Taken together, these findings deepen our understanding of how visualizations support logical reasoning and provide a model for improving analytical reasoning pedagogy.

## Introduction

Grasping the logical structure of arguments is foundational for higher-level reasoning and scholarly work. However, while one of the central aims of higher education is to equip students to comprehend argumentative texts and to reason clearly about them,^1^ the prerequisite skill of parsing such text into its logical components is rarely taught explicitly in universities. Moreover, standard university education provides at best modest improvements in students’ analytical-reasoning abilities.^2–4^ Since many students do not arrive at university with developed analytical skills, the benefits they can derive from readings and coursework are limited. What pedagogical approaches are most effective for cultivating these important skills? Here we investigate the effectiveness of a seminar-based undergraduate course in which students learned to analyze arguments in text by producing visualizations of their logical structure.

**Fig. 1:**
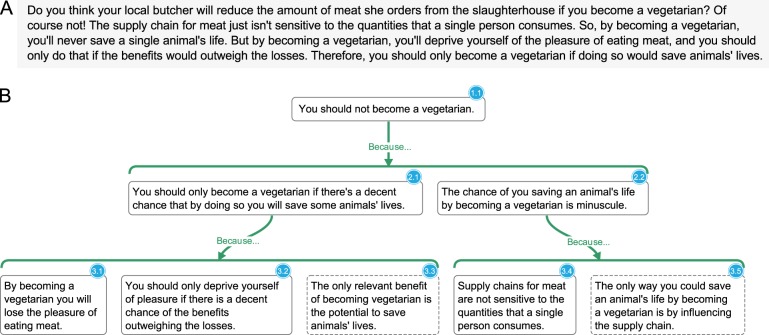
**a** Sample argumentative text. **b** Argument visualization for this text created using MindMup. Claims, the sentences contained in white boxes, are the fundamental units of argument visualizations. A reason is a set of claims grouped together underneath a horizontal green bracket labeled “because”. Reasons aim to raise one’s confidence in a conclusion. Claims are grouped together into a single reason when each claim must be plausible for either to support the conclusion; they are divided into separate reasons when they support a conclusion independently. An objection is a set of claims grouped together underneath a horizontal red bracket labeled “however”. Objections aim to lower one’s confidence in a conclusion and are constructed according to the same basic conventions. Dashed borders indicate premises which remain only implicit in the text (i.e., charitable assumptions required by the argument). This argument visualization shows that the conclusion (1.1) is supported by a reason which consists of two claims (2.1 and 2.2), each of which is supported by further reasons. The first of these reasons consists of three claims, one of which (3.3) remains implicit in the text; the second consists of two claims, only one of which (3.1) is explicitly stated in the text. The claims comprising each reason are perceptually unified beneath colored horizontal lines, encouraging the viewer to consider them jointly.^10^ Following conventions for graphical modeling, users can represent the ‘evidential strength’ of reasons/objections by increasing or decreasing the thickness of the connecting lines

Argument visualization traces to nineteenth-century logic pedagogy,^5^ and has been further developed in recent years.^6,7^ Argument visualizations are tree diagrams that illustrate logical relations in text by employing a combination of color and grouping cues to guide visual attention to salient elements^8,9^ and to bind elements that share a common function.^10^ Their hierarchical layout is intended to reflect the hierarchical structure of real arguments. These features make them helpful for both organizing and navigating complex argumentative texts and for communicating arguments transparently.^11^ Figure 1 provides an example of how argument visualization clearly exposes the logical structure in a sample of argumentative text.

The emphasis in this course on learning to produce argument visualizations is motivated by prior work on the benefits of generating explanatory visualizations for learning.^12–16^ Such benefits may be strongest when the visualization task encourages learners to continually check their explanations for completeness and coherence.^12,17^ Moreover, while verbal and written prose explanations provide opportunities for obfuscation, visual explanations may help to clearly convey what students understand.^16,18,19^ The availability of student-generated visualizations may also support the delivery of timely feedback from instructors that is tailored to each student’s level of understanding.^20,21^ Importantly, such graphical representations can also be readily shared and modified by others. As such, they can facilitate collaborative problem solving,^12,17,22^ especially when students lack vocabulary to describe their emerging understanding.^23,24^

The goal of the present study was to evaluate the hypothesis that effective training in argument visualization may lead to gains in students’ generalized analytical-reasoning skills, a possibility that researchers have recently begun to explore empirically.^6,25–27^ The present study advances prior work in three ways. First, our students train using real academic texts, rather than the highly simplified arguments often used in previous research, which may provide the necessary challenge and motivation to promote generalized improvements. Second, we use a reliable test of analytical reasoning with known psychometric properties,^28^ which correlates highly with real-world scholastic performance.^29^ Third, we include a control group, allowing us to estimate the degree to which improvements are due to our intervention as opposed to the generic effects of university education or maturation.

## Results

Students in our study participated in three activities each week. First, during seminar sessions, students worked in small groups (2–3 students) to create visualizations of arguments excerpted from contemporary analytic philosophy texts. Throughout these sessions, instructors circulated the room, providing students with assistance as needed. Second, students worked independently on problem sets which required them to construct argument visualizations from new philosophical texts. Third, students revised their argument visualizations in response to detailed feedback on their work-in-progress, which they received during weekly problem-set sessions. Only after this opportunity to revise their work in light of feedback did students submit their argument visualizations for assessment. Instructors then provided detailed and timely narrative feedback on students’ finished problem sets. Critically, instructors rarely provided students with explanations of the readings. Instead, motivated by prior work on the benefits of self-generated explanations for learning,^12–16,30–34^ all activities focused on guiding students as they actively generated their own representations of the texts.

According to weekly surveys that students completed when submitting their problem sets, students spent 5.52 h/week (SD = 1.32) working on problem sets, which they found to be difficult (4.1/5, SD = 0.73, where 5 is ‘extremely difficult’) and helpful in facilitating their understanding of the assigned readings (4.5/5, SD = 0.66, where 5 is ‘extremely helpful’).

### LSAT logical reasoning pretest

To assess whether this intensive training in argument visualization leads to generalized benefits for analytical reasoning, we administered equivalated LSAT Logical Reasoning forms to both seminar students and control students at the beginning and end of the semester (i.e., 85 days later). To control for possible differences between the forms, we randomly assigned 50% of students to form A as pretest and form B as posttest, reversing the order for the remaining students.

As a whole, students answered 16.8 questions correctly on the pretest (SD = 4.1). Pretest scores were higher in the control group (*M* = 17.4) than in the seminar group (*M* = 15.9), however this difference was neither large (*d* = 0.27) nor statistically reliable, *t*(114) = 1.4, *p* = 0.152.

### LSAT logical reasoning posttest

**Fig. 2:**
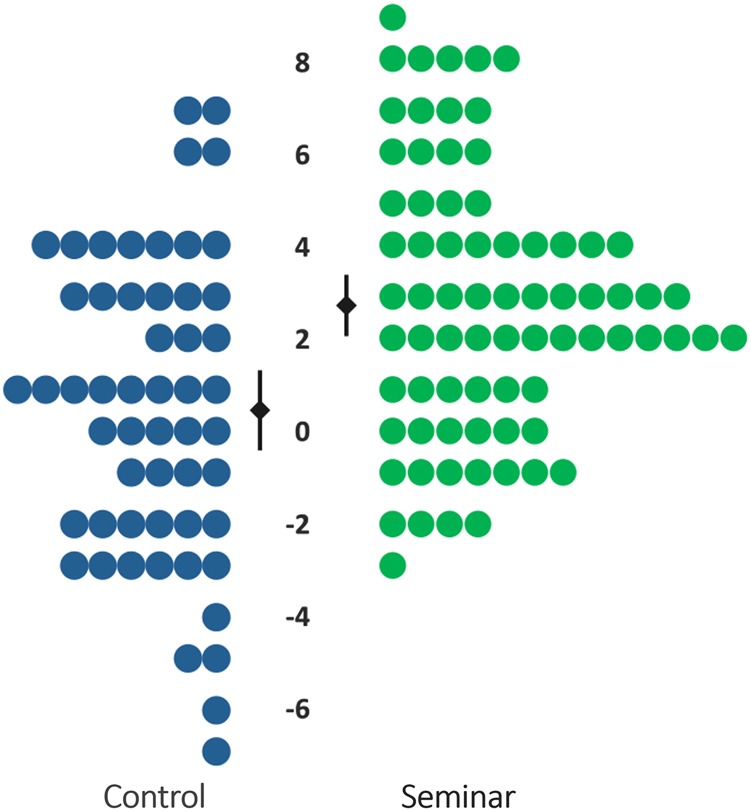
Change in LSAT logical reasoning test scores in each condition with 95% confidence intervals (*p* < 0.001)

Seminar students performed better on the posttest than they had on the pretest, *t*(104) = 9.6, *p* < 0.001, *d* = 0.77, 95% CI: [0.58, 0.95], answering an additional 2.6 questions correctly (SD = 2.8). Moreover, the degree of improvement exhibited by seminar students was greater than that exhibited by control students, *t*(159) = 4.3, *p* < 0.001, *d* = 0.71, 95% CI: [0.37, 1.04], whose scores did not change significantly between the beginning and end of the semester, *M*_change_ = +0.48 (SD = 3.2), *t*(55) = 1.1, *p* = 0.27, *d* = 0.11, 95% CI: [−0.09, 0.31] (Fig. 2).

To test whether the effect of group membership (seminar vs. control) remained reliable after controlling for prettest scores, we conducted an ANCOVA using group membership as the independent variable, pretest scores as the covariate, and improvement from pretest to posttest as the dependent variable. Having controlled for the relationship between pretest score and improvement, *B* = −0.40, 95% CI: [−0.49, −0.29], *p* < 0.001, we found a significant effect of membership in the seminar group, *B* = 1.5, 95% CI: [0.67, 2.34], *p* < 0.001, which accounted for a significant proportion of the variance in scores, *F*(1, 158) = 12.8, *p* < 0.001, $\eta _p^2$ = 0.075.

These results show that participating in the seminar led to meaningful improvements in students’ generalized analytical skills.

### Essay measures

We conducted an additional study to assess the impact of the seminar on students’ analytical skills and composition skills in the context of real academic assignments. In this study, control students were recruited from a large introductory philosophy lecture-based course offered in the same semester at Princeton University. For one unit of that semester, both seminar students and control students were assigned the same readings and wrote essays on the same topics. All essays from seminar students (*N* = 15) and a random sample of essays from control students (*N* = 44) were then evaluated by a grader who was blind to the identity of the author of each essay and the hypothesis under study. We found that seminar students (a) structured their essays more effectively, (b) presented the arguments more accurately, and (c) better understood the relevant arguments (Table 1) than did control students, *t*(57) = 2.9, *p* = 0.005, *d* = 0.87, 95% CI: [0.26, 1.48], with 81% of seminar essays earning a higher score than the mean-scoring control essay. In addition to scoring more highly on these dimensions, essays written by seminar students also received higher letter grades than those written by control students (Table 2).

**Table 1:** Descriptive statistics for essays from Seminar students (*N* = 15) and Control students (*N* = 44)

	Group	*M*	SD	SE
(a) Structure	Seminar	6.7	1.4	0.4
	Control	5.3	2.0	0.3
(b) Accuracy	Seminar	6.9	1.6	0.4
	Control	5.5	2.0	0.3
(c) Understanding	Seminar	7.5	1.1	0.3
	Control	5.8	1.8	0.3
(d) Grade point	Seminar	3.5	0.3	0.1
	Control	3.0	0.6	0.1

**Table 2:** Effect sizes (Cohen’s *d*) and *t*-tests for differences between Seminar and Control students’ essays

				95% CI for *d*	
	*t*	*p*	*d*	Low	High
(a) Structure	2.5	.014	0.76	0.16	1.36
(b) Accuracy	2.5	.017	0.74	0.13	1.33
(c) Understanding	3.2	.002	0.97	0.35	1.57
(d) Grade point	3.3	.002	0.97	0.36	1.58

## Discussion

We aimed to improve students’ generalized analytical-reasoning abilities by teaching them to visualize logical structures implicit in argumentative texts. We found that students’ abilities, as measured by parallel LSAT logical reasoning forms, improved substantially compared to students who did not take the seminar, *d* = 0.71, 95% CI: [0.37, 1.04]. Since actual LSAT administrations include two logical reasoning sections, the improvement in seminar students’ scores roughly corresponds to the difference between median scores at a US law school ranked in the top-10 and one ranked in the top-30. Moreover, Seminar students’ essays were more clearly written and evinced better understanding of the course readings than control students’ essays.

In sum, participating in our intensive argument-visualization seminar led to meaningful improvements in students’ analytical-reasoning skills relative to the baseline of receiving a standard university education at the same institution. This result is important because such skills are foundational for university-level study across the disciplines and improving them is the most commonly cited goal of undergraduate education.^35^

In anonymous end-of-semester surveys, students reported enjoying seminar and problem-set sessions, and many reported using argument visualization in coursework outside of the seminar. Between pretest and posttest, the number of students intending to major in philosophy increased by a factor of four in the seminar group but was stable in the control group. Students strongly agreed that the seminar improved their ability to construct and evaluate written arguments (4.9/5, SD = 0.36), to read and understand academic articles (4.9/5, SD = 0.36), and that their new skills would help them in other coursework (4.2/5, SD = 0.75). Across all iterations, students gave the seminar an overall course rating of 4.9/5.

Our findings resonate with a large literature showing that students often learn better when they play an active role in their own learning,^36–38^ such as by generating examples from their existing knowledge,^39,40^ asking probing questions of their instructors,^41^ devising their own methods for estimating a quantity,^42^ and explaining newly learned information to themselves.^30–34^

Some previous studies attempting to improve analytical-reasoning skills using argument visualization have reported similar effect sizes to those reported here.^26^ However, many of these studies did not provide comparisons to a control condition, and most relied on the California Critical Thinking Skills Test (CCTST) to measure improvements in students’ abilities. In recent studies where students completed both LSAT logical reasoning and CCTST forms, effect sizes were three times smaller with the LSAT than they were with the CCTST.^27^ While these recent studies succeeded in replicating previous results using the CCTST, they found only slight improvements with LSAT logical reasoning forms.

The precise explanation for this discrepancy between tests is unknown;^27^ however, the low psychometric quality of the CCTST may contribute. The CCTST exhibits low internal consistency and poor construct validity,^43–46^ making it difficult to give scores a clear interpretation. Moreover, the two forms of the CCTST are not statistically equivalent,^44,47^ and share identical or trivially modified items. Similar problems beset other common tests of critical thinking.^48^ By contrast, scores on LSAT logical reasoning forms exhibit high internal reliability,^28^ high stability across successive administrations,^49^ and correlate well with real-world scholastic achievement.^29^ As a result, findings based on LSAT logical reasoning forms provide significant additional evidence that a classroom intervention can produce large, generalized improvements in students’ analytical reasoning.

Our study had two distinctive pedagogical features. First, students learned how to visualize arguments contained in real scholarly texts, as opposed to highly simplified arguments. Second, their work was met with detailed and timely instructor feedback. Argument visualization provided the medium in which students could engage these texts and discuss their interpretations with the instructors and each other, and this was critically supported through effective pedagogy.^50^

The present findings do not fully disentangle the contributions of the use of argument visualization from the intensive and interactive nature of the course, as well as its explicit focus on argument analysis. While control students did participate in the standard Princeton University curriculum (which places a high value on rigorous analytical reasoning), they did not receive intensive training in non-visual argument analysis. Our current design therefore cannot speak to how much of the improvement in seminar students’ skills are due specifically to visualization. Disentangling these factors is a critical priority for future research, which will both advance our theoretical understanding of the underlying learning mechanisms and provide a clearer guide for curriculum development. Indeed, in order to more directly evaluate the contribution of training in argument visualization, per se, we are currently conducting a series of controlled laboratory experiments. In these studies, naive participants are instructed in argument analysis using either prose-based or diagram-based examples. All participants then read a series of brief argumentative texts, and answer multiple-choice questions assessing their ability to identify the logical structure latent in each. As they answer these questions, the diagram group inspects visualizations of the arguments, whereas the prose group views matched verbal descriptions. Insofar as the graphical elements of argument visualizations help students to analyze texts, we hypothesize that the visualization group will perform better and show greater improvement than the group who train on prose examples only. On the other hand, if the graphical elements do not enhance student comprehension, then we expect both groups to perform equivalently.

Taken together, our findings show that organizing good pedagogical practices (e.g., collaboration, feedback, constructive activities) around collaborative argument visualization leads to meaningful improvements in students’ analytical-reasoning skills and substantive understanding of course materials. We hope that future studies will investigate how students move beyond using argument visualizations to analyze existing prose and employ this technique to compose novel arguments. In the long run, findings from this line of inquiry will both deepen our understanding of how concrete visualizations support abstract reasoning and provide a model for improving analytical-reasoning pedagogy.

## Methods

### Participants

We offered a semester-long seminar as a part of Princeton University’s application-based Freshman Seminar Program. Between 2013 and 2017, 105 students participated in the seminar (60 female; *M*_age_ = 18.3, SD = 0.81), in seven semester-long courses of fifteen students each. During the same period, 56 control students were recruited from among freshmen at Princeton University (26 female; *M*_age_ = 18.1; SD = 0.67).

Due to institutional constraints, we could not randomize students into the seminar and control groups but had to use standard mechanisms for enrolling students; thus, our study was a quasi-experiment. Recruitment of control students focused on individuals who expressed interest in the seminar but were not enrolled due to limited space in the class. Thus, despite our use of a convenience sample, we were able to assemble a group of control students that did not differ significantly from the seminar group in their intended college majors at pretest, indicating that they took similar courses (other than the seminar). Moreover, pretest and self-reported SAT subject scores indicated that students in the two groups had comparable skills at pretest.

Control students did not receive explicit training in argument analysis using either visual or non-visual techniques. Control students either volunteered to participate without monetary compensation (*N* = 10) or were paid $20. Comparing paid and unpaid participants revealed no meaningful differences in test scores or outcome measures. We base our analysis on data from all control students and all students who enrolled in all iterations of the seminar. Self-reported SAT and ACT scores for our sample were consistent with admissions data,^51^ suggesting that our findings are relevant to students at selective colleges more generally. All participants provided informed consent and all study procedures were approved by the Princeton University IRB.

### Seminar sessions

**Fig. 3:**
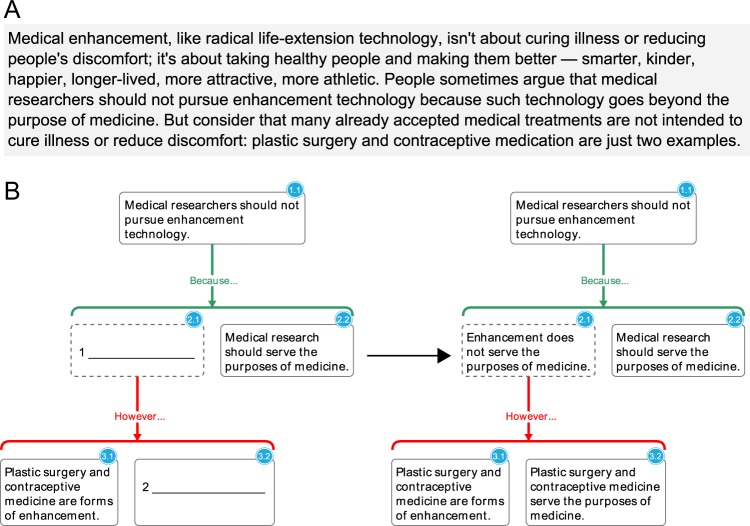
**a** Sample text. **b** Sample fill-in-the-blank exercise from an introductory problem set assigned early in the semester. Dashed borders mark claims which are implicit in the text (i.e., charitable assumptions required by the argument). Supporting reasons are represented by horizontal green brackets labeled “because”; objections are represented by horizontal red brackets labeled “however”

We trained students to practice close reading and argument analysis using web-based argument-visualization software. During class sessions, students worked in groups of two or three to analyze excerpts from philosophical texts and construct visualizations of the argument conveyed in each text. Unlike the simple example passages presented in Figs. 1 and 3, most texts used in the seminar were drawn from professional journals and books (e.g., Judith Jarvis Thomson’s “A Defense of Abortion,” Philippa Foot’s “Killing and Letting Die,” David Lewis’ “Are We Free to Break the Laws?,” and so forth.). To maintain an appropriate level of difficulty, these texts were sometimes adapted by the instructors. While students worked, three instructors circulated around the room, providing help or philosophical discussion when appropriate. A typical three-hour seminar was organized around three or four such argument analysis exercises and associated discussions.

### Problem sets

In weekly problem sets, students constructed argument visualizations based on excerpts from contemporary academic texts. Instructors encouraged students to collaborate on these assignments. At the beginning of the semester, problem sets consisted of simple fill-in-the-blank exercises (Fig. 3). After 4 weeks of training on pre-made exercises with progressively less scaffolding, students advanced to visualizing and analyzing arguments from scratch. Throughout the semester, additional support was provided in the form of weekly problem-set sessions hosted by the instructors, who provided general guidance on the current assignment, helped students to identify gaps in their understanding of the reading, and suggested ways for students to improve their work. Students then incorporated this feedback before submitting their work for assessment.

Students completed weekly surveys in which they reported how long they spent on the problem set, how difficult they found it, and to what degree it helped them to understand their readings. From week to week, feedback from students about the difficulty of the previous problem sets was used to calibrate the difficulty of the next problem set, with our target difficulty rating being 4/5. The course was designed to ensure that students practiced analyzing arguments for at least 10 h per week, including both classwork and homework.

In addition to coaching during the sessions, students received detailed and individualized written feedback on their problem sets every week, which indicated errors in students’ understanding of the texts as manifest in their argument visualizations. To convey a more accurate interpretation of the text, this feedback was often supplemented by a model solution. Common errors in representation include mistaking a premise for a support (and vice versa), representing co-premises as independent reasons (and vice versa), including unnecessary premises, and neglecting to represent important assumptions. During the semester, students were not informed of their grades in any form (e.g., alphabetical, numerical, checks/crosses) as we felt this would distract from the more valuable written feedback.

### Quantifying analytical-reasoning skills

To assess whether this intensive training in argument visualization leads to generalized benefits for analytical reasoning, we administered equivalated LSAT Logical Reasoning forms (Law School Admission Council; Newtown, PA) at the beginning and end of the semester (i.e., 85 days later). These forms are highly reliable (KR20 = 0.81, 0.79), have well-known psychometric properties,^28^ are heavily focused on argumentation skills, and are appropriately difficult for our sample. Furthermore, these forms include texts and pose questions very different to those presented during the seminar, making them an effective test of students’ ability to transfer their skills to a new context. To control for possible differences between the forms, we randomly assigned 50% of students to form A as pretest and form B as posttest, reversing the order for the remaining students.

### Assessing the quality of students’ essays

We stripped all identifying information from both seminar and control students’ essays. A grader blind to the hypothesis under study evaluated each essay using the following three-item scale:

1. How effectively structured is the essay?
2. How accurately presented are the relevant arguments?
3. How well does the student understand the relevant arguments?

Items were counterbalanced for order and rated on nine-point scales. Finally, essays were assigned letter grades according to the grader’s own standards for undergraduate essays.

Our three-item scale for rating the quality of students’ essays was highly consistent (*α* = 0.95), so the grader’s responses to the three items were summed to form overall essay scores.

### Code availability

In collaboration with the developers of MindMup, we created a free, open-source platform for argument visualization which is available at http://argument.mindmup.com. Readers who wish to learn more about using argument visualization in their own teaching may find useful resources collected at http://www.philmaps.com.

## Data Availability

The datasets generated and analyzed during the current study are available from the corresponding author upon reasonable request.
